# Epigenetic Modulation of Self-Renewal Capacity of Leukemic Stem Cells and Implications for Chemotherapy

**DOI:** 10.3390/epigenomes4010003

**Published:** 2020-03-01

**Authors:** Richard L. Momparler, Sylvie Côté, Louise F. Momparler

**Affiliations:** 1 Département de pharmacologie-physiologie, Université de Montréal, Montréal, QC H3C 3J7, Canada; 2 Service d’hématologie-oncologie, Centre de recherche, CHU Sainte-Justine, Montreal, QC H3T 1C5, Canada; sylvie_cote@yahoo.com (S.C.); louise.momparler@gmail.com (L.F.M.)

**Keywords:** leukemic stem cells, self-renewal, epigenetics, DNA methylation, histone methylation, 5-aza-2′-deoxycytidine, 3-deazaplanocin-A

## Abstract

Most patients with acute myeloid leukemia (AML) have a poor prognosis. Curative therapy of AML requires the complete eradication of the leukemic stem cells (LSCs). One aspect of LSCs that is poorly understood is their low frequency in the total population of leukemic cells in AML patients. After each cell division of LSCs, most of the daughter cells lose their capacity for self-renewal. Investigations into the role of Isocitrate dehydrogenase (IDH) mutations in AML provide some insight on the regulation of the proliferation of LSCs. The primary role of IDH is to convert isocitrate to alpha-keto-glutarate (α-KG). When IDH is mutated, it converts α-KG to 2-hydroxyglutarate (2-HG), an inhibitor of the TET pathway and Jumonji-C histone demethylases (JHDMs). The demethylating action of these enzymes removes the epigenetic gene-silencing markers, DNA methylation, H3K27me3 and H3K9me2 and can lead to the differentiation of LSCs. This enzymatic action is blocked by 2-HG in mutated IDH (mut-IDH) AML patients, who can be induced into remission with antagonists of 2-HG. These observations suggest that there exists in cells a natural enzymatic mechanism that uses demethylation to reverse epigenetic gene-silencing, leading to a loss of the self-renewal capacity of LSCs. This mechanism limits the proliferative potential of LSCs. Epigenetic agents that inhibit DNA and histone methylation exhibit a synergistic antineoplastic action on AML cells. It is possible that the therapeutic potential of this epigenetic therapy may be enhanced by demethylation enzymes, resulting in a very effective treatment for AML.

## 1. Introduction

**Figure 1:**
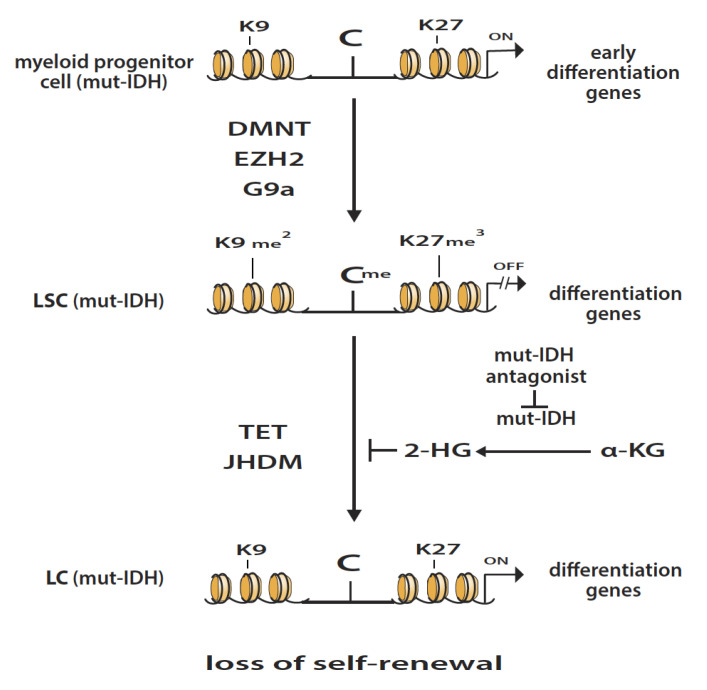
Transformation of myeloid progenitor cells with de novo mutation in IDH (mut-IDH) to leukemic stem cells (LSC). This transformation occurs when the genes that program differentiation are silenced by methylation by DNA methyltransferase (DNMT), and/or methylation of H3K27 and H3K9 by EZH2 and G9a, respectively. The LSC (mut-IDH) cells are characterized by a block in differentiation and have the capacity for self-renewal. The demethylation of DNA by the TET pathway and/or demethylation of H3K27me3 and/or H3K9me2 by JDHMs results in gene reactivation, induction of differentiation and loss of self-renewal. These alterations give rise to non-proliferating leukemic cells (LC (mut-IDH)). This latter process is inhibited in LSC (mut-IDH) cells due to the enzymatic conversion by mut-IDH of alpha-ketoglutarate (α-KG) to 2-hydroxyglutarate (2-HG), an oncometabolite that inhibits the demethylation action of the TET pathway and JHDMs, blocking their differentiation. Antagonists of mut-IDH inhibit the formation of 2-HG, reversing this block in differentiation, and can induce remissions in AML patients with mut-IDH.

Acute myeloid leukemia (AML) is characterized by the overproduction of abnormal myeloid cells which, when present in the bone marrow, suppress the production of normal blood cells [1]. Chemotherapy, consisting primarily of cytarabine and anthracyclines, can induce remission in many of the patients. However, when AML patients relapse, chemotherapy is less effective. There is an urgent need to develop more effective therapy. Leukemic stem cells (LSCs) are responsible for sustaining and propagating malignant disease, and thus are promising targets for therapy [2]. One of the major characteristics of AML cells is the block in differentiation which is due in part to epigenetic gene-silencing by DNA and histone methylation [3]. Chromatin analyses indicate that many of the genes marked with 5-methylcytosine in malignant cells also contain the polycomb repressive complex 2 marker (PRC2), histone H3 trimethylated lysine 27 (H3K27me3) [4,5]. Methylation of H3K27 is catalyzed by EZH2 histone methyltransferase (HMT), a subunit of PRC2 [6]. Functional analysis of the genes silenced by both DNA methylation and H3K27me3 in malignant cells indicate that a high percentage of these genes are involved in differentiation [7]. These reports suggest that epigenetic gene-silencing by DNA and histone methylation plays a major role in blocking the differentiation of malignant cells, including AML. These epigenetic alterations in cancer cells merit intensive analysis since they can identify key targets for chemotherapeutic intervention (see Figure 1).

## 2. Proliferative Potential of Leukemic Stem Cells

LSCs in AML represent a low-frequency subpopulation of leukemia cells that possess stem cell properties, a capacity for self-renewal and propagation of the malignant disease [2]. The LSC frequency is assessed in patients with AML by limiting dilution analyses in NOD/SCID/interleukin 2 receptor gamma chain null immunosuppressed mice [8]. The frequency of LSCs was observed to be in the range of one cell in 600 to >1000 cells. This very low frequency can be explained by the fact that, after each cell division of LSCs, most of the daughter cells lose their self-renewal capacity. This aspect is beneficial to the survival of AML patients because, theoretically, one LSC with a doubling time of about 24 h with no loss of self-renewal has the potential to expand up to 10^12^ cells in about 40 days. Since this explosive growth potential of AML cells is rarely observed in patients, there probably exists an intrinsic molecular mechanism that diminishes the full growth potential of LSCs.

## 3. Isocitrate Dehydrogenase Mutations in AML

Investigations into the role of isocitrate dehydrogenase mutations (mut-IDH) in AML give an important insight on the mechanisms that regulate self-renewal potential of LSCs [9]. Mutations of IDH1 and IDH2 are detected in about 20% of patients with AML [10]. The role of IDH is to catalyze the oxidative decarboxylation of isocitrate to alpha-keto-glutarate (α-KG). The catalytic activity of mut-IDH is modified so as to convert α-KG to 2-hydroxyglutarate (2-HG). Moreover, 2-HG acts as a competitive inhibitor of α-KG, resulting in the inhibition of the catalytic activity of the enzymes of the TET pathway and Jumonji-C domain histone demethylases (JHDMs). This inhibition by 2-HG prevents gene activation by DNA and histone demethylation in mut-IDH-AML cells and blocks their differentiation [11] (see Figure 1). For AML cells, the intrinsic catalytic activity of these two classes of enzymes can result in the random induction of their differentiation due to the reversal of epigenetic gene-silencing. This hypothesis can explain the loss of the self-renewal capacity of LSCs and their low frequency in patients with AML. These epigenetic alterations merit in depth investigation since they can give insight for the selection of targets for chemotherapeutic intervention in AML patients.

## 4. Epigenetic Enzymatic Functions That Favor the Development of Leukemogenesis

Key enzymes that have the potential to promote leukemogenesis when they are deregulated are DNA methyltransferase (DNMT) and the histone methyltransferases (HMTs), EZH2 and G9a. DNMT1 silences gene expression by the methylation of cytosine to 5-methylcytosine in the promoter region of genes [12]. EZH2 and G9a HMTs silence gene expression by the methylation of H3K27 to H3K27me3 and H3K9 to H3K9me2, respectively [13]. When myeloid progenitor cells are transformed to LSCs they can use DNMT, EZH2 and/or G9a to silence the genes that program differentiation (see Figure 1). This epigenetic silencing of these genes that program differentiation is one of the key events that occurs during leukemogenesis.

Epigenome analyses of AML cells indicate that they contain several thousand additional methylated CpGs compared to normal white blood cells [14]. The mechanisms that lead to this aberrant DNA methylation in cancer are not fully understood. It is possible that it is due to errors in the fidelity to copy the DNA methylation signature by DNMT1 [15] or by loss of fidelity due to mutations in DNMT3A [16]. Support that aberrant DNA methylation plays a very important role in leukemogenesis comes in the form of observations that the specific inhibitor of DNA methylation, 5-aza-2′-deoxycytidine (5AZA-CdR, decitabine), can induce complete remission in patients with AML [17]. The antileukemic action of 5AZA-CdR also correlates with its inhibition of DNA methylation [18]. In addition, 5AZA-CdR was also shown to reactivate tumor suppressor genes silenced by DNA methylation in malignant cells [12].

Support that EZH2 also plays an important role is leukemogenesis can be seen in the report that its inhibition of EZH2 by 3-deazaneplanocin A (DZNep) reduces the proliferation and induces differentiation of AML cells [19]. The overexpression of EZH2 in leukemic cells is a negative factor for overall survival in patients with myeloid malignancy [20]. There is a controversy surrounding the possible oncogenic action of EZH2 in lymphoid leukemia. Loss-of-function (LOF) mutations of EZH2 can lead to the development of T-cell leukemia, suggesting that it can also function as a TSG in malignant lymphoid cells [21]. It is also possible that this oncogenic action of EZH2 loss observed in T-cell leukemia may also be explained by the default function of EZH1 which replaces the role of EZH2 with a LOF mutation.

The presence of H3K9me2 gene-silencing marker in hematopoietic cells can also promote leukemogenesis. The methylation of this histone is catalyzed by G9a (EHMT2). The enhanced expression of G9a is involved in the proliferation of cancer cells [22]. In support of the belief that G9a can play an important role is leukemogenesis is the report that the inhibition of this HMT by BIX-01294 reduces the proliferation and induces the differentiation of AML cells [23].

## 5. Epigenetic Enzymatic Functions That Can Suppress Leukemogenesis

One of the most important enzyme functions that has the potential to suppress leukemogenesis is that of the TET family which can reverse gene-silencing by demethylation of DNA. In support of the anti-oncogenic action of TET2 is the report that AML cells with LOF mutations of this gene exhibit DNA hypermethylation and induction of leukemogenesis [24]. The DNA hypermethylation resulting from LOF mutations of TET2 blocks differentiation and favors the progression of AML [25].

Deregulation of members of the family of JHDMs also favors the development of leukemogenesis. IDH mutations impair histone methylation by the inhibitory action of 2-HG on JHDMs and result in a block of differentiation [11]. One of the key members of this family is KDM6A (UTX), the enzyme that demethylates H3K27me3. The demethylation of this histone gene-silencing marker leads to gene reactivation. LOF mutations of KDM6A have been observed in AML patients, suggesting that this enzyme can function as a TSG [26]. Mutations of KDM6A are present in about 46% of AML patients with a normal karyotype, suggesting that this gene has the potential to play a very important role in the development of leukemogenesis [26].

Another important member of the JHDM family is KDM3B (JHDM2B), the enzyme that demethylates H3K9me2. Demethylation of this histone gene-silencing marker also leads to gene reactivation. The knockdown of KDM3B in myeloid leukemia cells blocks granulocytic differentiation. [27]. The reduced expression of JHDM2B is observed in AML patients, suggesting that it can function as a TSG in leukemic cells [28].

## 6. Interaction between Different Epigenetic Gene-Silencing Mechanisms

TSGs that suppress leukemogenesis may be silenced by different epigenetic events. The major epigenetic gene-silencing mechanisms in AML cells are DNA methylation, H3K27me3 and H3K9me2. The interaction between these epigenetic alterations reveals the important role epigenetic gene-silencing markers play in the development of AML (Figure 1). When AML cells have two gene silencing markers, such as DNA methylation and H3K27me3, the effectiveness of therapy with 5AZA-CdR may be diminished due to its insufficient activation of TSGs that contain the second gene-silencing marker, H3K27me3 [29]. This limitation may be overcome by the using 5AZA-CdR in combination with a second epigenetic agent that diminishes the level of H3K27me3. Our studies on myeloid leukemia cells showed that 5AZA-CdR in combination with DZNep exhibits a remarkable synergistic antineoplastic effect and synergistic reactivation of genes [30,31]. These two epigenetic agents reverse gene-silencing by DNA methylation and H3K27me3. This observation suggests that this “double lock” mechanism of gene silencing plays a major role in the development of malignancy. 

Epigenome analysis revealed that about 25% of DNA methylated genes in cancer cells also contain H3K27me3 modification compared to only about 12% in normal cells [32]. These investigators also reported that 5AZA-CdR in combination with the EZH2 inhibitor GSK126 exhibited an additive in vitro growth inhibition of tumor cells and xenograft tumors of the prostate and breasts. In addition, TSGs were re-expressed more efficiently by the combined treatment than by a single agent treatment with 5AZA-CdR or GSK126. These observations indicate that this “double lock” mechanism of gene silencing exists in different types of cancer.

A second “double lock” mechanism of gene silencing can also occur by the presence of DNA methylation and H3K9me2 on the promoter of TSGs. Reactivation of these target genes would require the use of 5AZA-CdR in combination with an agent that reduces the level of H3K9me2, such as BIX-01294, which inhibits the methylation of H3K9 by G9a HMT [23,33]. We observed that 5AZA-CdR in combination with BIX-01294 exhibited synergistic antileukemic action on AML cells, as determined by colony assay [34]. This “double lock” mechanism may also play an important role in the development of AML. A similar “double lock” mechanism also exists in tumors. Sato et al. [35] reported that 5AZA-CdR in combination with the G9a inhibitor UNC0638 exhibited the synergistic activation of genes in tumor cells.

A third “double lock” mechanism can be caused by the presence of H3K27me3 and H3k9me3 in the promoter of TSGs. This type of inhibition can be reversed by a combination of DZNep, an inhibitor of EZH2, and BIX-01294, an inhibitor of G9a HMT. We observed that DZNep in combination with this HMT inhibitor of G9a (EHMT2) exhibited synergistic antileukemic action on AML cells, as determined by colony assay [34]. This result indicates that this “double lock” mechanism can also play an important role in the development of AML. A similar interaction was reported for breast carcinoma cells, as shown by a combination of an inhibitor of EZH2 and an inhibitor of G9a which exhibited a greater growth inhibition and gene activation than either agent alone [36].

Finally, a “triple lock” mechanism of gene silencing can also be due to the presence of 5-methylcytosine, H3K27me3 and H3K9me2 at the promoter region of TSGs. Gene reactivation, in this case, would require the three inhibitors that reverse three different types of epigenetic gene-silencing. The triple combination of 5AZA-CdR, DZNep and BIX-01294 exhibited greater antineoplastic action against the leukemic cells than any of the double combinations [34]. This “triple lock” mechanism of gene silencing may also play an important role in leukemogenesis.

**Figure 2:**
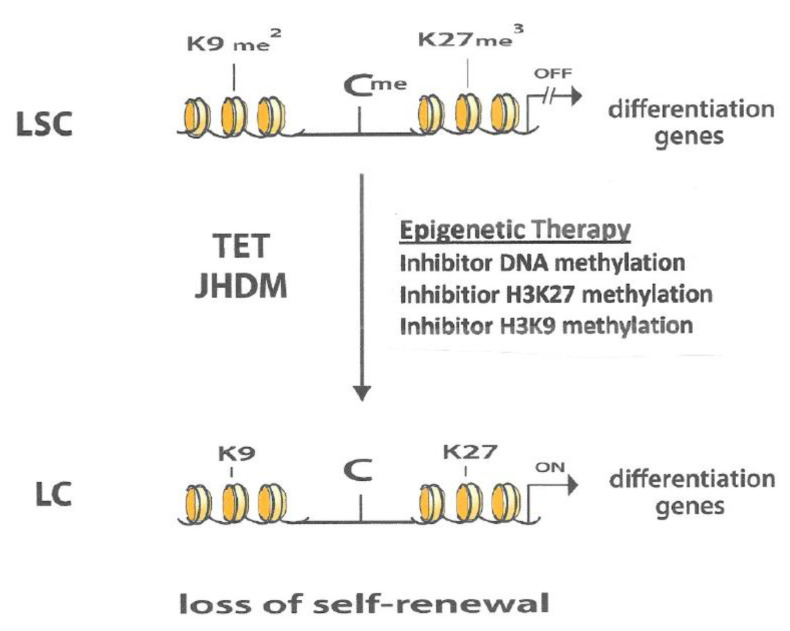
In leukemic stem cells (LSC) the genes that program differentiation are silenced by DNA methylation (Cme), methylation of H327 (Kme^3^) and/or methylation of H3K9 (Kme^2^). Treatment of LSCs with an inhibitor of DNA methylation, an inhibitor of H3H27 methylation and/or an inhibitor of H3K9 methylation leads to reactivation of genes that program differentiation and loss of self-renewal capacity of the leukemic cells (LC). The cellular intrinsic enzymatic demethylating action of the TET and JHDM can also lead to activation of differentiation and loss of self-renewal capacity of the LSC. It is possible that this intrinsic enzymatic mechanism can increase the effectiveness of the epigenetic therapy the treatment for AML.

These observations indicate that a “cross-talk” exists between the three different types of gene-silencing mechanisms providing a rationale for the use of two to three different epigenetic agents for the therapy of AML to reverse this gene-silencing (Figure 2). From a perspective of oncogenesis, the “cross-talk” between the three different gene-silencing mechanisms probably means that they interact in a positive manner to also promote leukemogenesis. A good understanding of these epigenetic interactions can lead to a better understanding of the major events that take place during the development of AML and can provide insights into potential key targets for chemotherapeutic intervention.

## 7. Conclusions

Studies of AML patients with IDH mutations reveal that DNA methylation and histone methylation play a major role in the development of leukemogenesis. In order for LSCs to retain their capacity for self-renewal they have to silence genes that program differentiation using, primarily, the epigenetic action of DNA methylation, H3K27me3 and/or H3K9me2. This gene suppression can be reversed by the demethylation of DNA by the TET pathway and the demethylation of H3K27me3 and/or H3K9me2 by JHDMs [9,10]. This intrinsic enzymatic action that exists in cells can lead to a loss of the self-renewal capacity of LSCs, limiting their proliferative potential, and may be responsible for their low frequency in AML patients [8].

It was a remarkable discovery that mut-IDH-AML cells produce the oncometabolite 2-HG that inhibits the TET pathway and JHDMs. This action blocks the differentiation of LSCs by the prevention of TSG reactivation via the inhibition of DNA and histone demethylation [9,10]. The importance of this interaction is shown by the induction of remissions in AML patients with mut-IDH by antagonists of 2-HG [9,10]. These observations clarify the important role of these mechanisms of epigenetic gene-silencing in the development of leukemogenesis. It also indicates that epigenetic agents that inhibit DNA and histone methylation have an interesting potential for the therapy of AML. This assumption is supported by the observation that the combination of 5AZA-CdR and DZNep exhibit synergistic antineoplastic activity against AML cells [30,31]. The chemotherapeutic potential of this novel epigenetic therapy may also be enhanced by the intrinsic enzymatic action of the TET pathway and JHDMs present in cells (Figure 2).

The selection of the most active epigenetic agents is essential to improve the effectiveness of therapy of AML. The epigenetic agent 5AZA-CdR is one of the most potent due to its high selectivity to target cancer cells, its preferential upregulation of genes with promoter DNA methylation, and its preference for targeting genes that are silenced in cancer [35]. In addition, preclinical studies of 5AZA-CdR demonstrated it to be a more potent antileukemic agent than cytosine arabinoside or 5-azacytidine [37]. In order to benefit from the full chemotherapeutic potential of 5AZA-CdR, there is a need to optimize the dose schedule of this epigenetic agent [38]. The S-phase specificity of 5AZA-CdR is one of the major reasons why the antineoplastic action of this analogue is very dependent on its dose schedule [39]. For a second epigenetic agent for use in combination with 5AZA-CdR, we declare our preference to be DZNep due to the remarkable antileukemic synergy of these two agents when used in combination [30,31]. In addition, we observed that DZNep is a more potent antileukemic agent than several inhibitors that target the catalytic site of EZH2 [40]. Clinical studies on AML patients will determine if the combination of 5AZA-CdR and DZNep is more effective than the standard chemotherapy used to treat this hematological malignancy.

The remarkable antileukemic synergy observed between 5AZA-CdR and DZNep was first observed in human HL-60 myeloid leukemia cells [30]. An important question is whether this novel epigenetic therapy has the potential to be effective against other types of malignancies. This type of antineoplastic interaction was also observed in murine L1210 lymphoid leukemia cells [30] and in human A549 lung carcinoma cells [41]. These observations suggest that these epigenetic interactions can also occur in other cancers. This signifies that the combination of 5AZA-CdR and DZNep may also have the potential to be effective therapy for other types of malignancies.

## Abbreviations

AML	Acute myeloid leukemia
LSC	Leukemic stem cell
IDH	Isocitrate dehydrogenase
α-KG	Alpha-keto-glutarate
2-HG	2-Hydroxyglutarate
JHDM	Jumonji-C domain histone demethylase
mut-IDH	Mutated Isocitrate dehydrogenase
TET	Ten-Eleven-Translocation
PRC2	Polycomb repressive complex 2
EZH2	Enhancer of zeste 2 polycomb repressive complex 2 subunit
DNMT	DNA methyltransferase
5AZA-CdR	5-Aza-2′-deoxycytidine
DZNep	3-Deazaneplanocin-A
H3K27me3	Histone 3-lysine 27-trimethylated
H3K9me2	Histone 3-lysine 9-diimethylated
